# A principled machine learning framework improves accuracy of stage II colorectal cancer prognosis

**DOI:** 10.1038/s41746-018-0057-x

**Published:** 2018-10-02

**Authors:** Neofytos Dimitriou, Ognjen Arandjelović, David J. Harrison, Peter D. Caie

**Affiliations:** 1 School of Computer Science, University of St Andrews, St Andrews, KY16 9SX UK; 2 School of Medicine, University of St Andrews, St Andrews, KY16 9TF UK

**Keywords:** Cancer microenvironment, Colorectal cancer

## Abstract

Accurate prognosis is fundamental in planning an appropriate therapy for cancer patients. Consequent to the heterogeneity of the disease, intra- and inter-pathologist variability, and the inherent limitations of current pathological reporting systems, patient outcome varies considerably within similarly staged patient cohorts. This is particularly true when classifying stage II colorectal cancer patients using the current TNM guidelines. The aim of the present work is to address this problem through the use of machine learning. In particular, we introduce a data driven framework which makes use of a large number of diverse types of features, readily collected from immunofluorescence imagery. Its outstanding performance in predicting mortality in stage II patients (AUROC = 0:94), exceeds that of current clinical guidelines such as pT stage (AUROC = 0:65), and is demonstrated on a cohort of 173 colorectal cancer patients.

## Introduction

Colorectal cancer (CRC) is the third most common cancer worldwide and the leading cause of death among gastrointestinal tumours.^1,2^ Annually, there are 1.4 million new cases and more than half a million of deaths worldwide.^1^ A typical CRC diagnosis requires the evaluation of histopathological slides from a biopsy or resected specimen by a pathologist.^3,4^ Subsequent to a positive diagnosis, prognosis is assessed based on the tumour-node-metastasis (TNM) staging system.^5^ The TNM stage is considered by far one of the best predictors of CRC^6^ and as a consequence, statistics specific to the stage primarily guide therapy. However, stages that exhibit higher variability in survival, encounter greater uncertainty. Stage II patients do not experience nodal (N) or distant (M) metastasis of their cancer and so only the depth of local invasion (T) is reported under TNM staging. Stage II CRC patients countenance an estimated 20% of 5-year poor prognosis, and 35% of 10 years poor prognosis.^7,8^ Nevertheless, there are no definite criteria for selecting which, if any, stage II patients should undergo adjuvant chemotherapy with different trials reaching inconsistent conclusions.^9,10^ It is therefore imperative to improve upon the prognosis of stage II CRC patients to better aid clinical guidance, reduce the survivability variance, and consequently, ameliorate treatment research.

Histopathological review of patient tissue sections by a pathologist remains subjective and thus suffers from inherent inter- and intra-observer variability. This affects TNM staging, especially due to the introduction of criteria within the staging guidelines, which are harder to standardize.^11,12^ Nevertheless, this has a greater negative impact when reporting features independent of TNM that may aid in determining stage II patients with a higher risk of disease specific death.^11,12^ One such feature is histological grading, or equivalently differentiation, currently within the core data set of international reporting guidelines for CRC.^3,13^ Despite attempts to maintain consistency in reporting this feature, such as moving from a three-tiered system down to two tiers, reproducibility issues persist.^3,13^ Other promising histopathological features for further stratifying stage II CRC patients include lymphatic vessel invasion and tumour budding.^14–16^ However, they are currently listed within non-core data items,^3^ despite consistent demonstration of their prognostic significance. This has been attributed to the high observer variability and hence, methodological shortcomings of quantifying these features in a standardized manner.^17,18^

Both medical practice and research are moving towards a more nuanced approach in clinical decision-making. Pathology is now embracing the era of digitization with a multitude of interdisciplinary studies employing techniques from fields such image analysis, machine learning (ML) and deep learning.^19–22^ The use of these techniques markedly increases efficiency and efficacy compared to traditional methods, while removing the subjectivity imposed by the human pathologist.^23–25^ Moreover, multiplexed detection of target proteins is becoming more commonplace in pathology research through wider adoption of immunofluorescence (IF). Data collected through IF provide a multi-dimensional representation of the tumour micro-environment with each biomarker co-registered to the same physical coordinates in the tissue. In addition, utilizing specific antibodies to visualize histopathological features overcomes common issues of reporting from H&E stained tissue, such as retraction artefact confounding lymphatic vessel invasion and high density immune infiltrate obscuring tumour buds.^17,18^ Therefore, employment of techniques from the aforementioned fields on IF data have the potential to exploit multidimensional data, ranging from morphometric to spatial characteristics of selected histopathological features, and aid in improving prognosis for stage II CRC patients.

The present work builds upon previous efforts in the field,^26^ which make use of image analysis for the extraction of histopathological features (such as nuclear grade, tumour budding and lymphatic vessel invasion, cellular shape, size, texture, etc.), a priori known or expected to be salient, and simple statistical techniques for the subsequent inference. In particular, we describe a principled and data driven framework which uses modern machine learning to predict the survival outcome for a stage II CRC patient from a large number of histopathological features.

## Results

### Full feature set based prognosis

Each baseline classifier’s hyperparameter values were learnt by maximizing the corresponding average area under the receiver operating characteristic curve (AUROC) on the validation data corpus. Table 1 summarizes the results. The average AUROC across all classifiers was found to be 0.89 both for 5- and 10-year prognosis. One-way analysis of variance (ANOVA) and Tukey’s honest significance difference test (THSD) showed no statistical significance between classifiers for 10-year prognosis. The only statistically significant difference is that between naïve Bayes (NB) and logistic regression (LR)-based approaches for 5-year prognosis (ANOVA *p* value < 0.01, THSD *p* value < 0.003).

**Table 1:** Average AUROC and standard deviation (for *n* = 200) of trained classifiers on the training set using 20-times repeated tenfold cross-validation

	LSVM	RSVM	LR	RF	KNN	NB
5 year	0.89 ± 0.12	0.89 ± 0.13	0.91 ± 0.12	0.89 ± 0.13	0.88 ± 0.12	0.86 ± 0.14
10 year	0.89 ± 0.13	0.89 ± 0.12	0.91 ± 0.119	0.90 ± 0.13	0.89 ± 0.13	0.88 ± 0.12

*LSVM* linear kernel SVM*, RLSVM* radial basis function kernel SVM

**Fig. 1:**
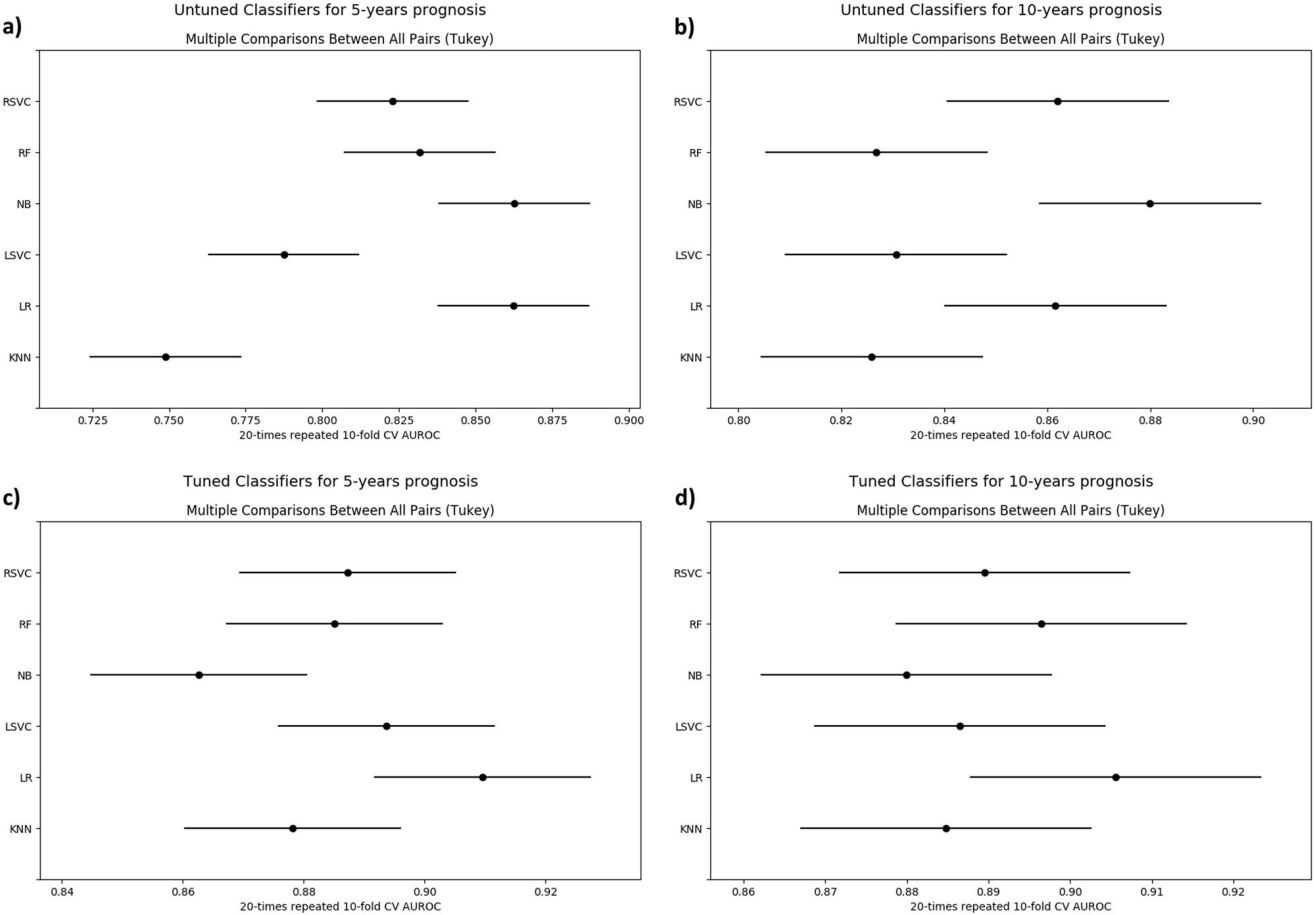
Tukey’s significance difference test. No hyperparameter learning was employed in the experiments corresponding to the plots **a** and **b**, in contrast to **c** and **d**

To demonstrate the importance of model selection, we also compared the performance of all classifiers using hyperparameter values, which were learnt as described in the previous section, and with the a priori set hyperparameters values as in the existing literature. As expected, using the latter approach a drop in the average AUROC was observed both for 5- and 10-year prognosis, to respectively 0.82 (approximately 8.0% drop) and 0.85 (approximately 4.5% drop). The results are visualized in Fig. 1.

### Reduced feature sets

#### Feature selection

**Fig. 2:**
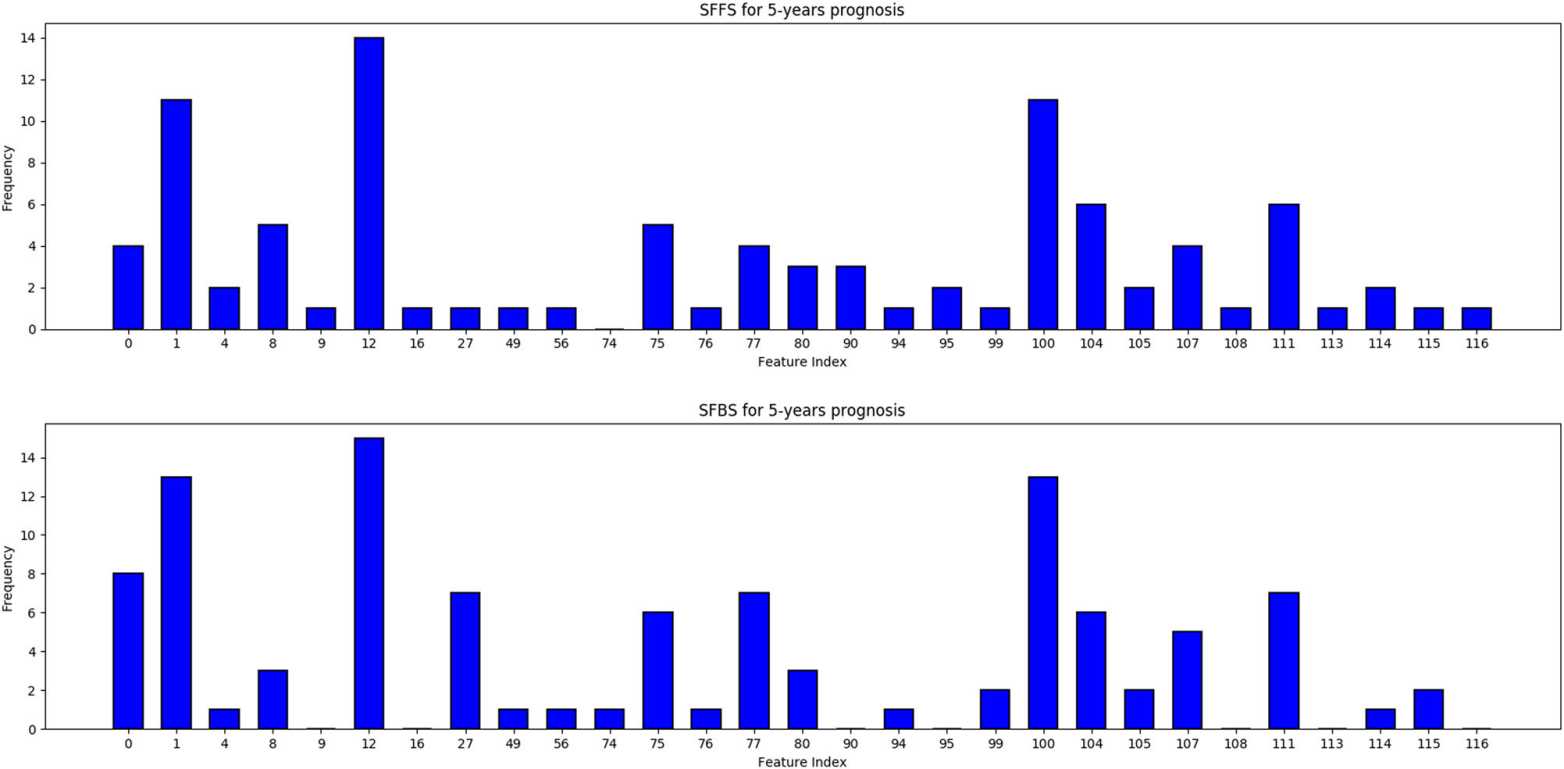
Frequency of occurrence of each feature from the 20 runs of SFFS and SFBS each for 5-year prognosis. Only features with at least one occurrence are shown for clarity

**Fig. 3:**
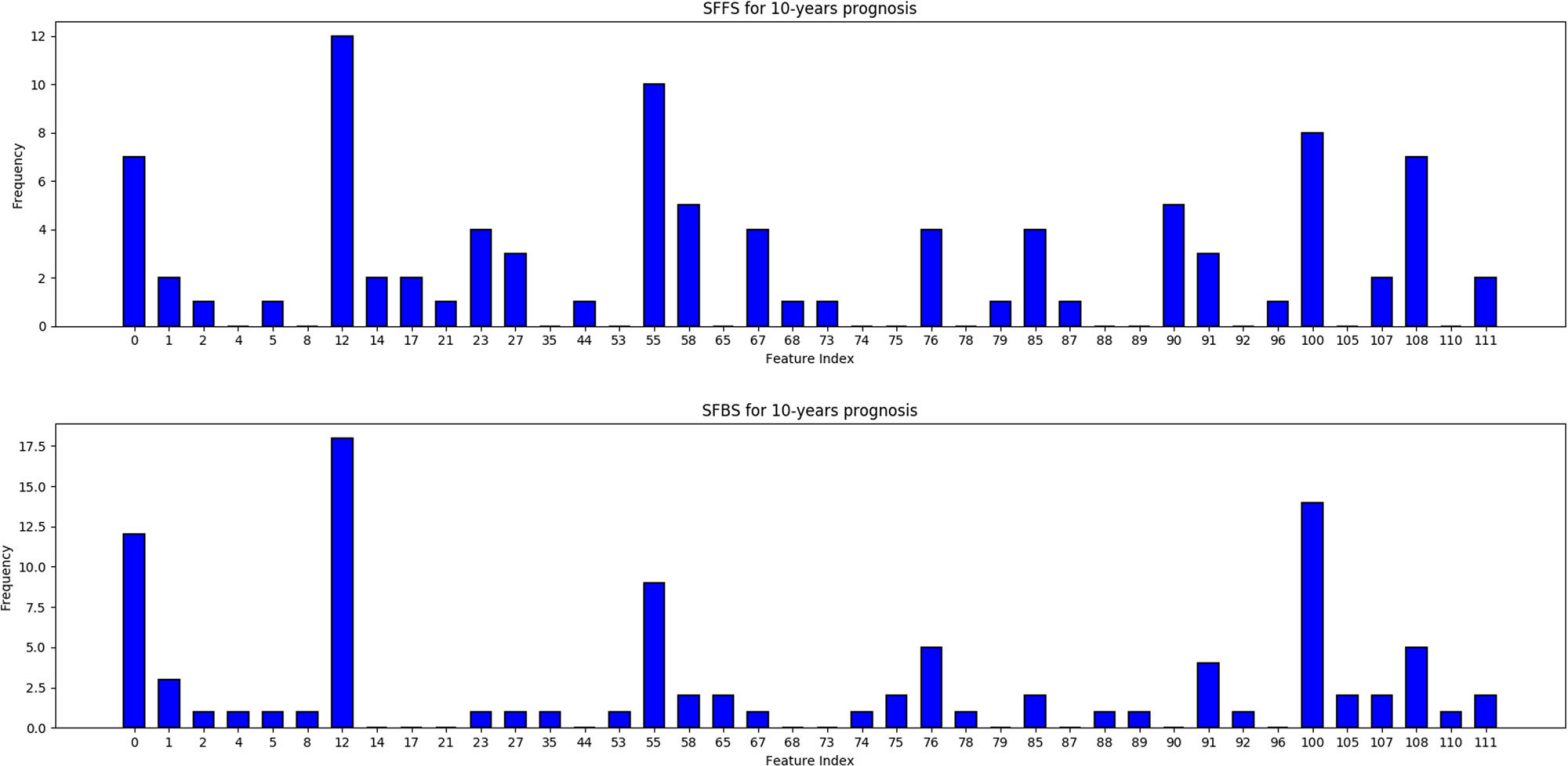
Frequency of occurrence of each feature from the 20 runs of SFFS and SFBS each for 10-year prognosis. Only features with at least one occurrence are shown for clarity

The evaluation of each subset of features was performed by tenfold cross-validation on the training data. To reduce outcome variability caused by stochastic effects we adapt the method proposed by Dune et al.^27^. In particular, we performed sequential floating forward search (SFFS) and sequential floating backwards search (SFBS) 40 times using different random partitions, each time retaining the feature subset that achieved the best performance. Following aggregation—see Figs. 2 and 3—the subsets from SFFS and SFBS were combined and features ordered based on the frequency of occurrence. Starting with an empty set, features were added in an incremental fashion based on their average AUROC rank, estimated through 20-times repeated tenfold cross-validation. The subset of features that achieved the highest averaged AUROC was selected for each prognostic term, as summarized in Table 2.

**Table 2:** Features of significance to both prognosis terms, and those which were specific to a particular term; seven and six features were used for 5 and 10-year prognosis, respectively

	#	Features
Unique to 5-year prognosis	4	Nuclei in tumour mean DAPI intensity, number of CK objects with no associated nuclei, sum area of vessels, average DAPI intensity (tumour area)
Unique to 10-year prognosis	3	Nuclei in tumour mean D240 intensity, mean compactness of tumour glands, number of PDCs
Common to both prognoses	3	Nuclei in tumour bud mean DAPI intensity, tumour gland relative area (%), sum area of vessels

*CK* pancytokeratin, *PDCs* poorly differentiated clusters

#### Experiments

We followed the same approach to classifier training, model selection, and evaluation as in the previous section. The sole difference is that instead of the full feature set, for this set of experiments a reduced set of selected features (as described previously) was used.

**Fig. 4:**
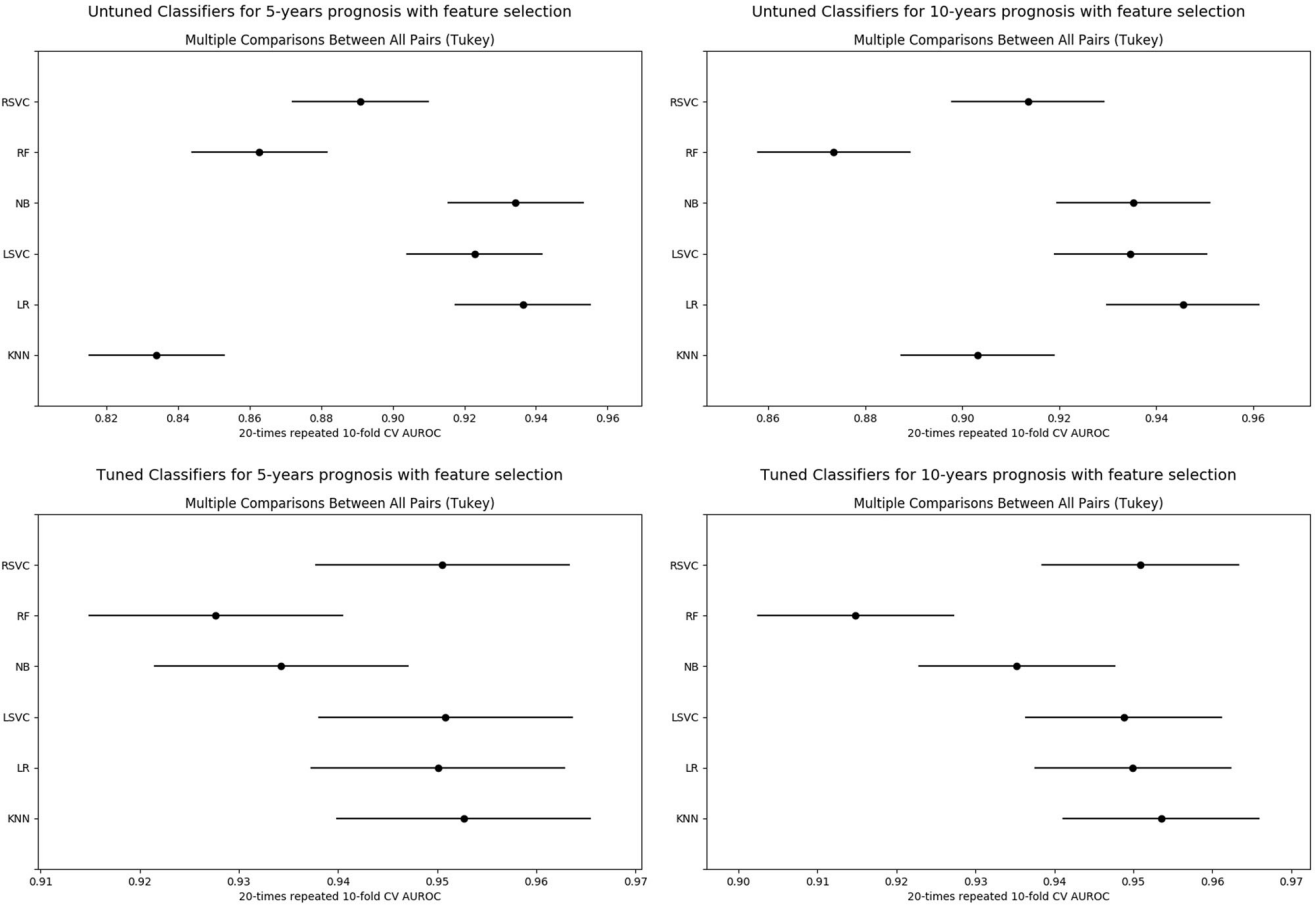
Tukey’s significance difference test. No hyperparameter learning was employed in the experiments corresponding to the plots **a** and **b**, in contrast to **c** and **d**

As expected, we observed a significant improvement in performance already at the coarsest level of analysis, with the average AUROC across classifiers reaching 0.94, both for 5- and 10-year prognosis. In line with our previous findings, no statistically significant difference was observed between different classifiers, except for the inferiority of random forest (RFs) for 10-year prognosis (ANOVA *p* < 0.0001, THSD *p* < 0.01). Just as in the previous set of experiments, our data driven approach to hyperparameter selection was always found to effect a statistically significant improvement over their being set a priori; see Fig. 4.

### Final testing

We started by examining training set performance of different classifiers using 20-times repeated tenfold cross-validation. It can be readily seen that classifiers trained on the subset of features selected by SFFS and SFBS performed better, as illustrated in Tables 1 and 3. Though simple, the best performing classifier was found to be KNN-based classifier (with the Minkowski distance metric) both for 5-year (*k* = 36) and 10-year prognosis (*k* = 28).

**Table 3:** Average AUROC and standard deviation (for *n* = 200) of each trained classifier using only features selected by SFFS and SFBS

	LSVM	RSVM	LR	RF	KNN	NB
5 years	0.95 ± 0.08	0.95 ± 0.08	0.95 ± 0.08	0.93 ± 0.11	0.95 ± 0.08	0.93 ± 0.10
10 years	0.95 ± 0.08	0.95 ± 0.08	0.95 ± 0.08	0.92 ± 0.10	0.95 ± 0.07	0.94 ± 0.09

The experiments were performed by 20 times repeating tenfold cross-validation on training data.

**Fig. 5:**
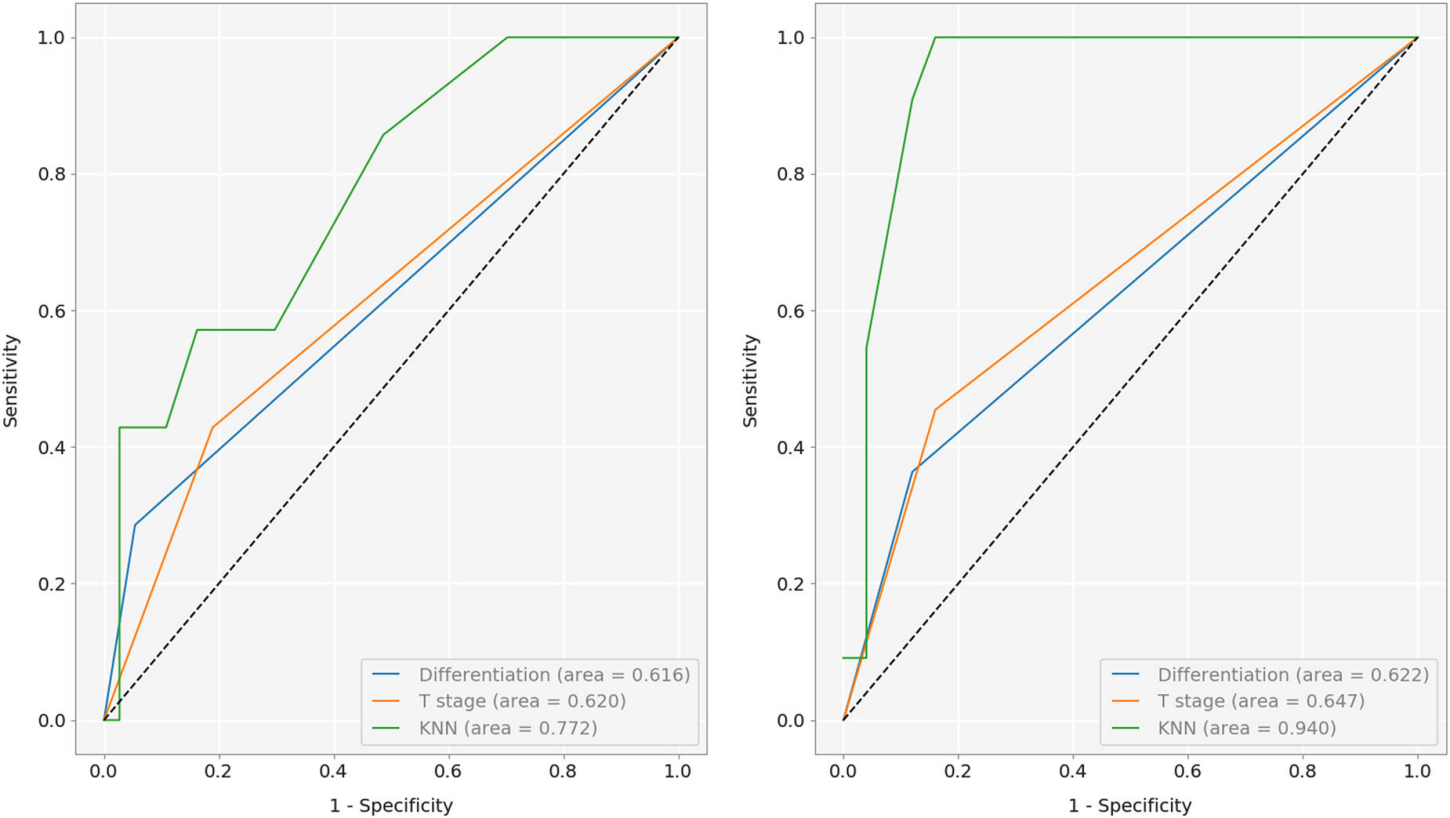
ROC curves for the two prognostic terms of interest

**Fig. 6:**
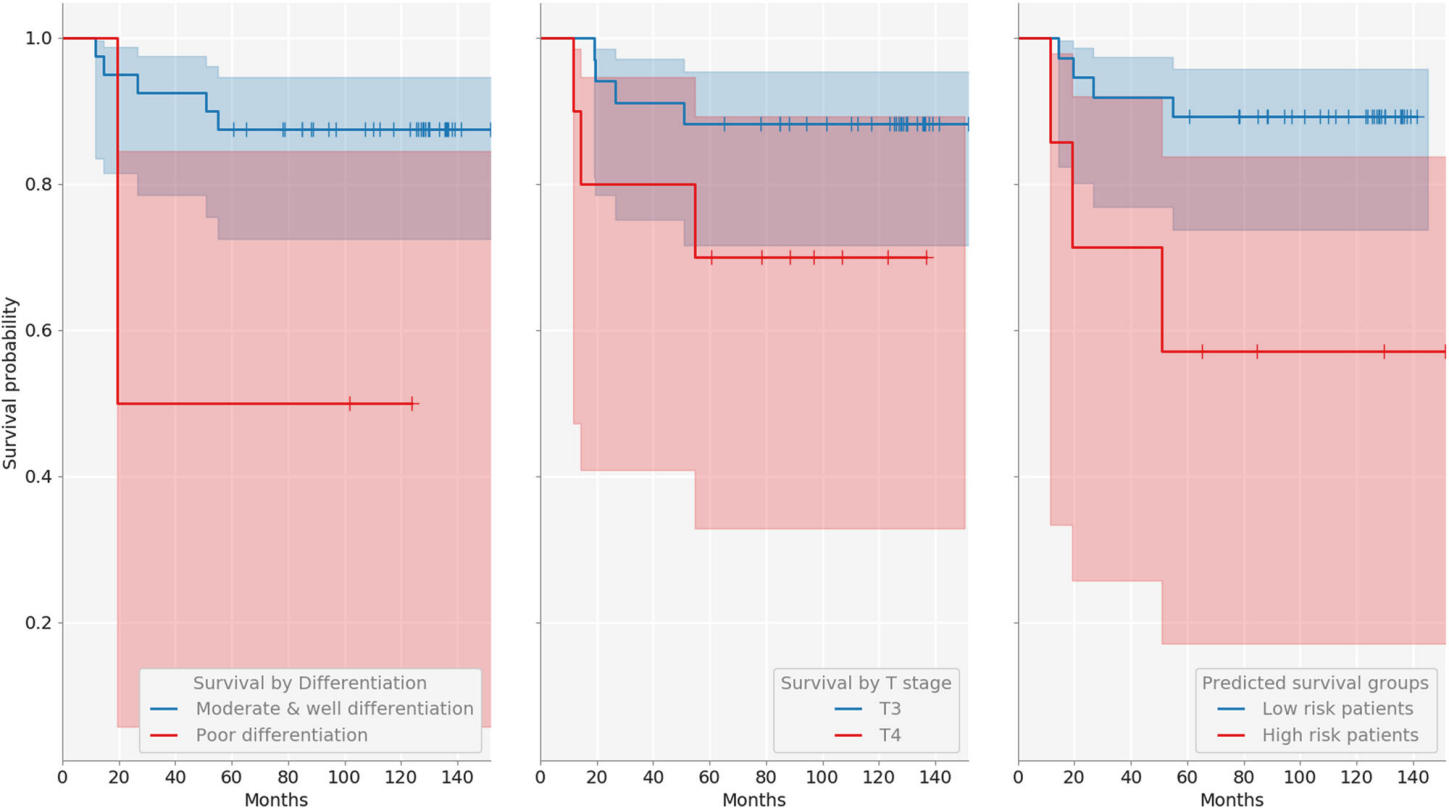
KM curves for 5-year prognosis

**Fig. 7:**
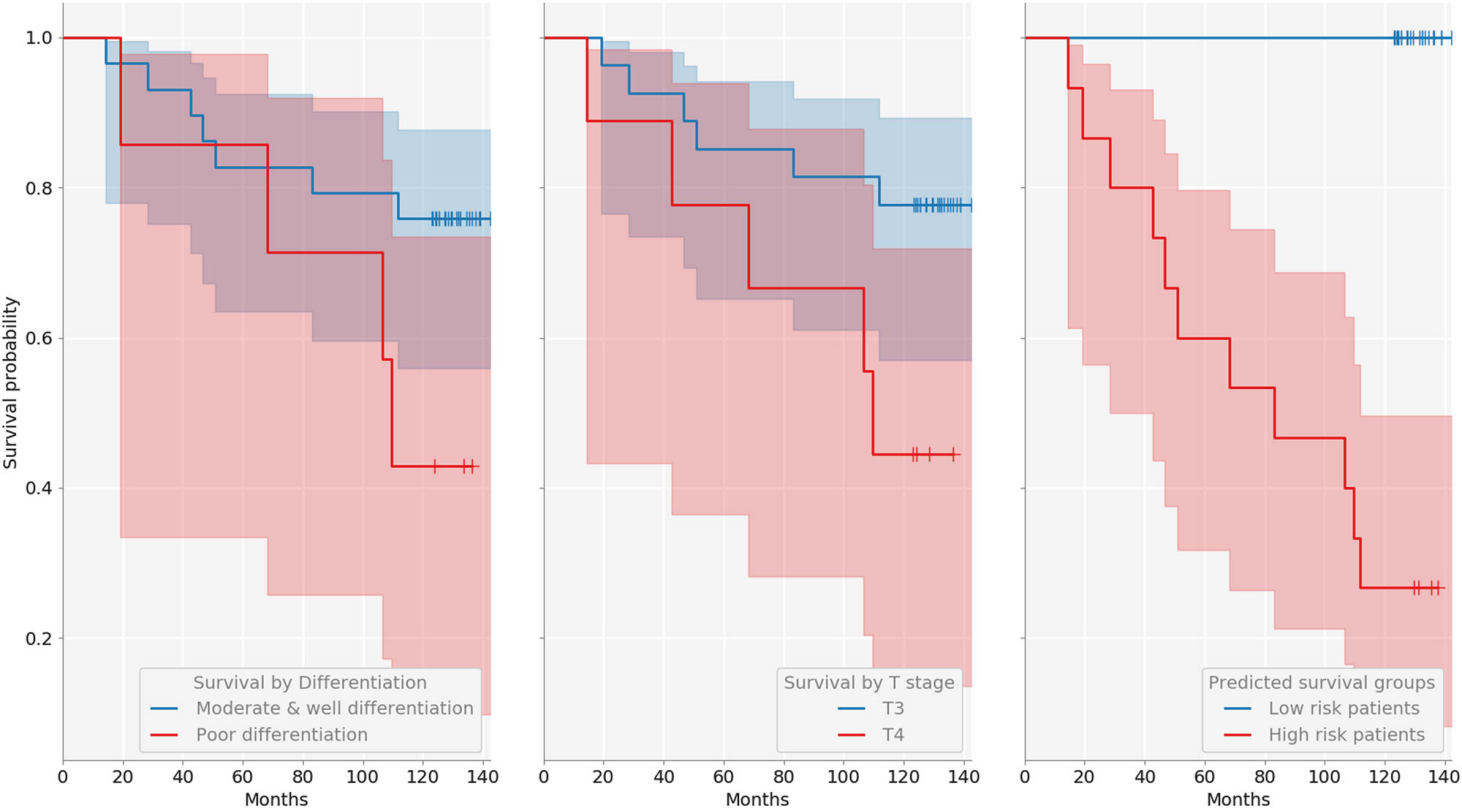
KM curves for 10-year prognosis

Kaplan-Meier (KM) survival curves were employed to visualize the difference in survivability between the predicted prognosis groups, and the log-rank test used for objective quantification thereof. For 5-year prognosis, our KNN-based approach achieved the AUROC of 0.77, effecting a good separation patients into high and low-risk (*p* value < .02). On 10-year prognosis, the classifier achieved the AUROC of 0.94, significantly outperforming the current clinical gold standard of pT stage (AUROC of 0.65), and even better separation between high- and low-risk patients (log-rank test *p* < .0001). The sensitivity of 42.9%, specificity of 89.2%, and accuracy of 81.8% were achieved for 5-year prognosis, and the sensitivity of 100%, specificity of 84%, and accuracy of 88.9%, for 10-year prognosis. The differentiation (poor/moderate vs. good) and T stage discrimination (T3 vs. T4) results are summarized in Figs. 5, 6 and 7, as well as in Table 4.

**Table 4:** Summary of low vs. high risk patient separation results

	Differentiation (5/10 year)	T stage (5/10 year)	KNN (5/10 year)
Specificity	0.95/0.88	0.82/0.84	0.89/0.84
Sensitivity	0.39/0.36	0.43/0.46	0.43/1.00
Accuracy	0.84/0.72	0.75/0.72	0.82/0.89
AUROC	0.62/0.62	0.62/0.65	0.77/0.94

## Discussion

CRC is a highly heterogeneous disease, which limits the prognostic accuracy of the TNM staging system or the reporting based on individual features such as tumour budding,^28^ or lymphatic vessel invasion and density.^29^ Prior work on the use of automated image analysis and ML applied to other types of cancer has focused on parameters solely from tumour cells.^20,21^ However, the evidence from an increasing number of studies suggests that the tumour micro-environment is just as informative,^30–32^ which motivated us to use information not only from tumour nuclei but also from numerous hierarchical features such as texture, morphology, fluorescence intensity, and spatial relationships across the micro-environment of the invasive margin. Hence, we introduced a carefully crafted ML based framework capable of nuanced prediction of survival for stage II CRC patients. Our methodology was shown to outperform significantly the current gold standard in the form of pT staging. Specifically, our method achieved AUROC of over 77 and 94% for 5 and 10-year prognoses respectively, compared to pT stage, which stratifies patients with the AUROC of approximately 62% both for 5- and 10-year prognosis, and the differentiation, which achieves the corresponding AUROC of approximately 62 and 65%, respectively. Moreover, we demonstrated high interpretability of the proposed approach, allowing clinicians to gain new insight by identifying prognostically the most salient features.

Confirming findings from prior empirical research as well as one of the premises of the present work, our experiments demonstrated that a diverse set of characteristics of the entire micro-environment have a prognostic value. This explains the outstanding performance of our method and the major improvement on the current state of the art which focuses on a single aspect thereof (usually tumour cells). DAPI intensity within the nuclei of tumour buds was consistently found to carry the greatest prognostic weight, which too agrees with previous empirical findings—cells within more invasive and mesenchymal tumour buds have increased plasticity and gene expression,^15,33^ which effects an increase in DAPI intensity. Furthermore, in the present study, this feature was highly correlated with parameters associated with tumour bud nuclei morphometry, whereby features linked to larger and more irregular shaped nuclei (such as found in more aggressive poorly differentiated cancer cells) were associated with poorer prognosis. This phenomenon would further explain an increase in the DAPI intensity within parameters describing other tumour subpopulations and which are reported parameters from the model. Tumour gland nuclear morphometry, also found to be of major prognostic importance, has also been identified in the past.^34,35^ Other selected features included known histopathological features such as the number of PDCs,^36^ the number and area of lymphatic vessels,^29^ and the shape and area of tumour glands.^37,38^

It is interesting to observe and comment on our finding that certain features were specifically associated with a particular prognostic term. Having looked at this in detail, we found high correlation between these features, within specific survival terms, and outcomes, suggesting that the features are not specific to set survival times per se but are rather associated with poorer outcomes. For example, the number of small pan cytokeratin positive objects with no associated nuclei was found to be an important feature for 5-year survival. On the other hand, the number of PDCs was found to be an important prognostic feature for 10-year survival. Nevertheless, both were highly correlated with the number of tumour buds.

Digital pathology is becoming more common in the clinical workflow, with recently, Glasgow and Oxford hospitals committing to a fully digital workflow. The digitization of pathology will allow the embedding of image analysis and AI solutions into a pathologist's routine practice. Fully automated workflow, such as the one presented here, allows results to be reported to the patient in a shorter time frame while freeing up more of a pathologist's large workload. Studies such as these add to the body of work exemplifying proof of concepts, which use image analysis and AI for cancer pathology. In order for automated image analysis and AI to be translated into the clinic further regulatory approved validation studies must be applied utilizing large patient cohorts sourced from multiple institutions.

In summary, the present work made several important contributions: (i) a principled framework for data driven ML based precise prognosis of stage II CRC cancer outcomes, (ii) significantly better performance than the current state of the art, (iii) clinical insight into the disease, and (iv) demonstrated the general potential of modern ML in digital pathology and health care more broadly. Following the highly promising results reported herein, our future work will focus on the application of computer vision and ML directly on histopathological tissue slides, so as to avoid the loss of information associated with ‘atomization’ of the process^39^ effected by human driven feature extraction and the subsequently applied learning from these. Additionally, in order to increase the potential for clinical adoption of the developed methodologies, it will likely be of interest to consider how the results should be presented to the clinician.^40,41^

## Methods

Our experimental data were obtained from tissue samples of 180 Scottish patients who had been diagnosed with CRC and who underwent surgical resection, with a minimum follow-up of 11.5 years. Patients that succumbed within 5 days of the surgery were excluded to ensure that surgical complications did not contribute to the cause of death, as were the three patients that received therapy due to potential effects on the relevant micro-environment and hence survival.^42^ Table 5 summarizes the key clinical and demographic characteristics of the cohort.

**Table 5:** Summary of patient cohort statistics

Number of patients		173
Age (years)		
	Range	62.5 ± 33.5
	Median	67
Gender		
	Male	86 (50%)
	Female	87 (50%)
T Stage		
	TX	1 (1%)
	T1	6 (3%)
	T2	7 (4%)
	T3	122 (71%)
	T4	37 (21%)
N Stage		
	N0	163 (94%)
	N1	8 (5%)
	N2	1 (1%)
	N3	1 (1%)
M Stage		
	MX	9 (5%)
	M0	161 (93%)
	M1	3 (2%)
Site		
	Rectum	56 (32%)
	Colon	117 (68%)
Differentiation		
	Undetermined	3 (2%)
	Poor	25 (14%)
	Moderate	138 (80%)
	Good	7 (4%)

The use of tissue samples was approved by the East of Scotland Research Ethics Service (13/ES/0126). Further ethical clearance was not required as the acquired data was anonymized. For more detailed patient information please see the previous work of Caie et al.^26^

### Features

The digitization of the tissue samples, and subsequent quantification and extraction of histological features were part of the work completed by Caie et al.^26^ Both are briefly described hereunder but interested readers should refer to Caie et al.^26^ and the corresponding supplementary document for a more thorough overview.

Tissue samples were prepared for multiplex immunofluorescence with pan cytokeratin and D2-40 antibodies, along with DAPI stain for the detection of epithelial cells, lymphatic vessels, and cell nuclei. The invasive front was manually identified through the pan cytokeratin channel of each whole-slide image captured at 40× magnification. Fifteen evenly spaced high-resolution (200× magnification) images were captured across the invasive front of each sample. Regions of interest (ROIs) (including stroma, tumour glands, invasive tumour subpopulations, lymphatic vasculature, and cell nuclei) were detected and segmented from each imported image using Definiens AG image analysis software package. Each ROI was described by a collection of morphometric, spatial, and fluorescence related characteristics associated with each patient, resulting in 123 histopathological features (independent variables); for further detail see Supplementary Document.

For each patient, pathological and demographic features were collected as well. The former set comprises the level of differentiation, site of primary tumour, and the corresponding disease stage, and the latter the patient’s age, gender, survival status at multiple clinically relevant follow-up intervals, and (where applicable) time until death. Except for the survival status, which was the dependent variables of interest in the present work, the remaining features were used for the analysis of experimental results, and not for the actual learning and prediction.

### Data preparation

We followed the standard approach to algorithm training and evaluation, by splitting the cohort dynamically into non-overlapping training, validation (or development), and test subsets. In particular, data were first randomly (with stratification) split into two, 70 and 30%, the latter being the test subset. Using tenfold cross-validation, the former, large subset was in each iteration of the process further randomly split into training and validation subsets.

It is worth noting that, given the key aim of the present work, while the evaluation corpus contain only stage II patients, we decided to include differently staged patients in the training corpus. Our hypothesis was that in spite of not being the target population for our prediction, useful pathological patterns could be learnt from this data too, allowing a degree of interpolation to take place. Stratified sampling was employed in order to maintain the prognosis distribution of each cohort as a means of countering the imbalanced nature of our data, and thus avoid class under-representation.^43^ Lastly, features were normalized to zero mean and unity variance.

### Baseline classification and performance assessment

The problem at hand was formalized as a binary, supervised classification task, whereby the prediction was that of a good or bad prognosis, i.e. survived or not, respectively. We adopted several well-understood baseline classifiers, with different underlying assumptions (explicit or implicit) and mathematical underpinnings. In particular, we compared classifiers based on support vector machines,^44^ RFs,^45^ *k*-nearest neighbours (KNN),^46^ NB,^47^ and LR.^48^ In an effort to capture performance adequately on a highly imbalanced data set, the AUROC^49^ is adopted as the primary performance measure. In addition, for the sake of consistency with related work and ease of comparative analysis, we also report specificity and sensitivity, and accuracy.

### Model selection

The capability of a model to represent information, as well the efficiency its learning is governed by a number of parameters. These parameters, referred to as hyperparameters, need to be set prior to training. However, finding the optimal or close to optimal set of hyperparameter values is challenging. The commonly used and probably the simplest approach, in the form of a grid search has limited applicability due to its intractability for complex models. A random search over predefined ranges of hyperparameters often produces better results while being computationally less demanding.^50^ However, both techniques are naïve as they do not take into account historical patterns.

Sequential model based global optimization (SMBO) techniques adopt a more sophisticated approach, approximating the possibly computationally expensive fitness function with a simpler surrogate.^51^ Different SMBO approaches optimize different criteria which then guide the surrogate of the fitness function. The one adopted herein is tree-structured Parzen estimator (TPE), which optimizes the so-called ‘expected improvement’. Conceptually, TPE initially behaves like a random search, subsequently refining the search so that hyperparameter values associated with poor performance are not re-visited.^51,52^ This process is guided probabilistically, using suitable densities or distributions associated with the type of hyperparameter. Those used in the present work are summarized in Table 6. Finally, as the loss function we used the negated AUROC resulting from tenfold cross-validation, averaged over 20 independent runs and using 500 iterations.

**Table 6:** The search space of each classifier based on the distributions over its hyperparameters (n.b. *F* denotes feature count; for biased categorical distributions, tuples (*p*_*s*_, *v*) designate the sampling probability and the value assigned)

Classifier	Hyperparameter	Distribution	Values
SVM, linear kernel	C	Log-uniform	[ln (1e−5), ln (1e2)]
	Class weight	Categorical	Balanced or none
SVM, RBF kernel	C	Log-uniform	[ln (1e−5), ln (1e2)]
	Gamma	Log-uniform	[ln (1e−3), ln (1e3)]
	Class weight	Categorical	Balanced or none
LR	Type of penalty	Categorical	L1 or L2
	C	Log-uniform	[ln (1e−5), ln (1e2)]
	Class weight	Categorical	Balanced or none
RF	Number of trees	Log-uniform integer	[10, 1000]
	Criterion	Categorical	Gini or entropy
	Maximum features	Biased categorical	(0.2, √F), (0.1, ln F), (0.1, F), (0.6,U(0, F))
	Maximum depth	Biased categorical	(0.1, 2), (0.1, 3), (0.1, 4), (0.7, none)
	Bootstrap	Categorical	True or False
	Class weight	Categorical	Balanced or none
KNN	K	Log-uniform integer	[1, 50]
	Weights	Categorical	Uniform, or Euclidean distance
	Metric	Categorical	Balanced or none
	P	Categorical	Balanced or none

### Feature selection

In order to address potential problems associated with the so-called curse of dimensionality, which becomes of increasing concern with a large number of features, we examined the use of dimensionality reduction in the context of the problem at hand.^53,54^ In particular, motivated by their successful use in the existing literature^55^ we employed SFFS and SFBS,^55–57^ which respectively perform recursive removal or addition of features in an attempt to improve a specific metric, until the desired reduction in the feature number is attained.

### Code availability

Full code is available from the authors upon request.

## Electronic supplementary material

Supplementary Information

## Data Availability

The data used in this work is available from the authors upon reasonable request.

## Electronic supplementary material

**Supplementary information** accompanies the paper on the *npj Digital Medicine* website (10.1038/s41746-018-0057-x).
